# Neoadjuvant immunotherapy for resectable primary liver cancer (Review)

**DOI:** 10.3892/ol.2025.15204

**Published:** 2025-07-23

**Authors:** Qingsong Deng, Minglian He, Leida Zhang, Yuzhang Wu

**Affiliations:** 1 Institute of Immunology, Third Military Medical University, Army Medical University, Chongqing 400038, P.R. China; 2 Army Institute of Hepatobiliary Surgery, Southwest Hospital, Third Military Medical University, Army Medical University, Chongqing 400038, P.R. China

**Keywords:** neoadjuvant immunotherapy, hepatocellular carcinoma, hepatectomy, liver transplantation, radiofrequency ablation

## Abstract

Primary liver cancer (PLC) is the third leading cause of cancer-associated mortality worldwide. The most effective curative treatment for liver cancer is radical hepatic resection; however, >50% of patients experience relapse within 2 years. Immune checkpoint inhibitors (ICIs) are effective adjuvant treatments for resectable hepatocellular carcinoma (HCC) following hepatic resection, as they decrease postoperative recurrence risk and prolong patient survival. Clinical trials aim to evaluate the safety and feasibility of neoadjuvant immunotherapy and indicate that ICIs are tolerated and more effective in decreasing local cancer recurrence and metastasis compared with standard neoadjuvant or adjuvant targeted therapies. For resectable intrahepatic cholangiocarcinoma, almost all neoadjuvant therapy regimens involve chemotherapy combined with immunotherapy, but these treatments are available only to those participating in ongoing clinical studies. The present review presents the most relevant efficacy and safety results of completed and ongoing clinical trials and discusses challenges associated with the administration of ICIs for PLC in the neoadjuvant setting. The use of neoadjuvant immunotherapy in patients before liver resection, transplantation, radiofrequency ablation or similar procedures has been investigated primarily through exploratory clinical trials. Neoadjuvant immunotherapy is a promising and safe perioperative treatment for resectable HCC and has acceptable efficacy. Extensive clinical trials with definitive support for this approach are needed to justify its clinical application.

## 1. Introduction

Primary liver cancer (PLC) is the third most common cause of cancer-associated mortality globally (1). PLC primarily comprises hepatocellular carcinoma (HCC), accounting for 75–85% of cases, and intrahepatic cholangiocarcinoma (ICC), accounting for 10–15% of cases. The 5-year survival rates are 18 (2) for patients with HCC and 8% (3) for patients with ICC. A total of 12.1% of patients with HCC in China survive for 5 years (4). Orthotopic liver transplantation (OLT) or radical hepatic resection are the standard and most effective curative treatments for early-stage HCC (5,6); treatment selection is contingent upon liver dysfunction and the extent of the tumor (7). Surgical intervention is key for the long-term survival of patients with PLC, as the 5-year survival rate is >50% among Chinese patients who undergo surgery (8). However, the efficacy of the procedure is limited by a low initial rate of R0 resection, which occurs in 15–30% of cases; it is also limited by the high postoperative recurrence rate, with 5-year tumor recurrence and metastasis rates ranging from 40 to 70% (9). Of patients with HCC, ~54% experience HCC recurrence within 22 months of primary resection and active postoperative management (10,11). For original tumors <2 cm in diameter, the recurrence rate approaches 70% (12), markedly limiting HCC treatment efficacy and impacting survival rate.

Sorafenib, a small molecule-targeted antiangiogenic drug, has been used to prevent HCC recurrence (13–16). Notably, the STORM trial demonstrated that postoperative antiangiogenic therapy does not prevent recurrence or prolong survival in patients with HCC (17). Immune checkpoint inhibitors (ICIs) exhibit notable clinical efficacy in patients with disease progression following tyrosine kinase inhibitor (TKI) therapy and may increase survival rates when used in conjunction with a cytotoxic T lymphocyte antigen 4 (CTLA-4)-blocking antibody (18–20). Lygidakis and Parissis (21) demonstrated that combining hepatectomy with targeted transarterial regional immunotherapy and chemotherapy notably extends the survival time compared with that of the hepatectomy group. In addition, compared with local chemotherapy, combined local immunotherapy exhibits superior survival and responses (22). Nevertheless, the outcomes associated with local chemotherapy combined with immunotherapy and systemic chemotherapy surpass those associated with systemic immunotherapy alone (23). Gardini *et al* (24) explored the application of tumor-infiltrating lymphocytes (TILs) combined with IL-2 for adjuvant immunotherapy, however, no notable differences were observed in the 1-, 3- or 5-year disease-free survival rates. Chiang *et al* (25) reported that supercharged natural killer (sNK) cells exhibit markedly greater cytotoxicity against well-differentiated HCC compared with untreated or IL-2-stimulated primary NK cells. sNK-cell-based immunotherapy may serve as a neoadjuvant or adjuvant treatment for poorly differentiated HCC.

Immunotherapy for HCC has been extensively studied, and several clinical trials are underway (26,27). Phase 1/2 studies have demonstrated the promising clinical efficacy of ICIs, such as programmed cell death protein 1 (PD-1), programmed death ligand 1 (PD-L1) and CTLA-4 inhibitors, as second-line treatments for HCC (18,20), including unresectable and advanced HCC (28–30); these ICIs have good response rates, and 12.9% (95%CI, 6.7–19.1%) patients achieve a pathological complete response (pCR) (31). The primary immunotherapeutic agents in clinical trials are nivolumab (18), pembrolizumab (30), durvalumab (32), atezolizumab (33), tremelimumab (34) and ipilimumab (35). Immunotherapy has potential value in the prevention and treatment of the postoperative recurrence of HCC, especially for patients with a high recurrence risk, such as patients with microvascular invasion, multiple nodules or tumors >5 cm in diameter (36). By activating the immune system to clear residual cancer cells, immunotherapy may markedly prolong recurrence-free survival (RFS) and overall survival (37). Atezolizumab and bevacizumab have been used as adjuvant therapy for high-risk HCC following surgery and notably decrease the risk of recurrence (12-month RFS rate, 78 vs. 65% in the control group) (38). A phase 3 randomized trial (trial no. NCT03847428) is in progress to evaluate the safety and efficacy of ICIs for adjuvant treatment of HCC. Previous studies indicate that postoperative adjuvant immunotherapy is beneficial (38–41). Owing to its clinical efficacy, the Food and Drug Administration (FDA) of the USA has approved adjuvant immunotherapy for several types of cancers, such as non-small cell lung cancer and melanoma (42,43).

Neoadjuvant therapy is a systemic treatment administered prior to the surgical removal of resectable liver cancer. Neoadjuvant therapy can be a systemic treatment involving a single class of drugs; the combination of multiple drugs in neoadjuvant therapy exacerbates adverse reactions in patients. Therefore, neoadjuvant immunotherapy has advantages over adjuvant therapy. The hepatectomy may damage systemic immunity. Elevated levels of alarm proteins, including damage-associated molecular patterns, and peripheral cytokines, such as IL-8 and IL-6, contribute to local and systemic inflammation (44,45). This inhibits cell immunity, hinders the generation and activation of T cells and may further limit the efficacy of adjuvant immune checkpoint blockade therapy (46,47). Resection and destruction of the tumor microenvironment and associated draining lymph nodes impact immunotherapy efficacy. Compared with neoadjuvant immunotherapy, PD-1-targeting agents may promote T cell expansion at these lymph nodes, and surgical damage to normal lymphatic vessels may diminish the efficacy of adjuvant immunotherapy. Preoperative immunotherapy can enhance antitumor immunity, which may be due to the activation of T cells in response to new tumor antigens when the tumor is still in the body; in adjuvant therapy, the new antigens only originate in micrometastases from the liver cancer lesions, which may lead to decreased immune activation (48,49). A preclinical model reported that, compared with postoperative treatment only, PD-1 blockade before surgery improved the survival rate and enhanced the activation of tumor-specific CD8^+^ T cells in mice (50). Moreover, data from small cohort studies show that when preoperative immunotherapy is administered, clonal T cell amplification is enhanced (48,51). Candidates for surgery may be more sensitive to immunotherapy compared with late-stage patients because surgical candidates have a stronger immune system. Additionally, immunotherapy is typically administered following numerous other treatment strategies that further tax the immune system, including combination therapy.

At present, most neoadjuvant therapies for PLC are targeted therapies and combined with immunotherapy (52). For resectable liver cancer, most surgeons still perform direct surgical resection. However, certain clinical research results have demonstrated the role of neoadjuvant immunotherapy in PLC (53–55). Thus, the present study reviewed ongoing clinical trials and neoadjuvant monoimmunotherapy for resectable PLC.

## 2. Methodology

**Figure 1.**
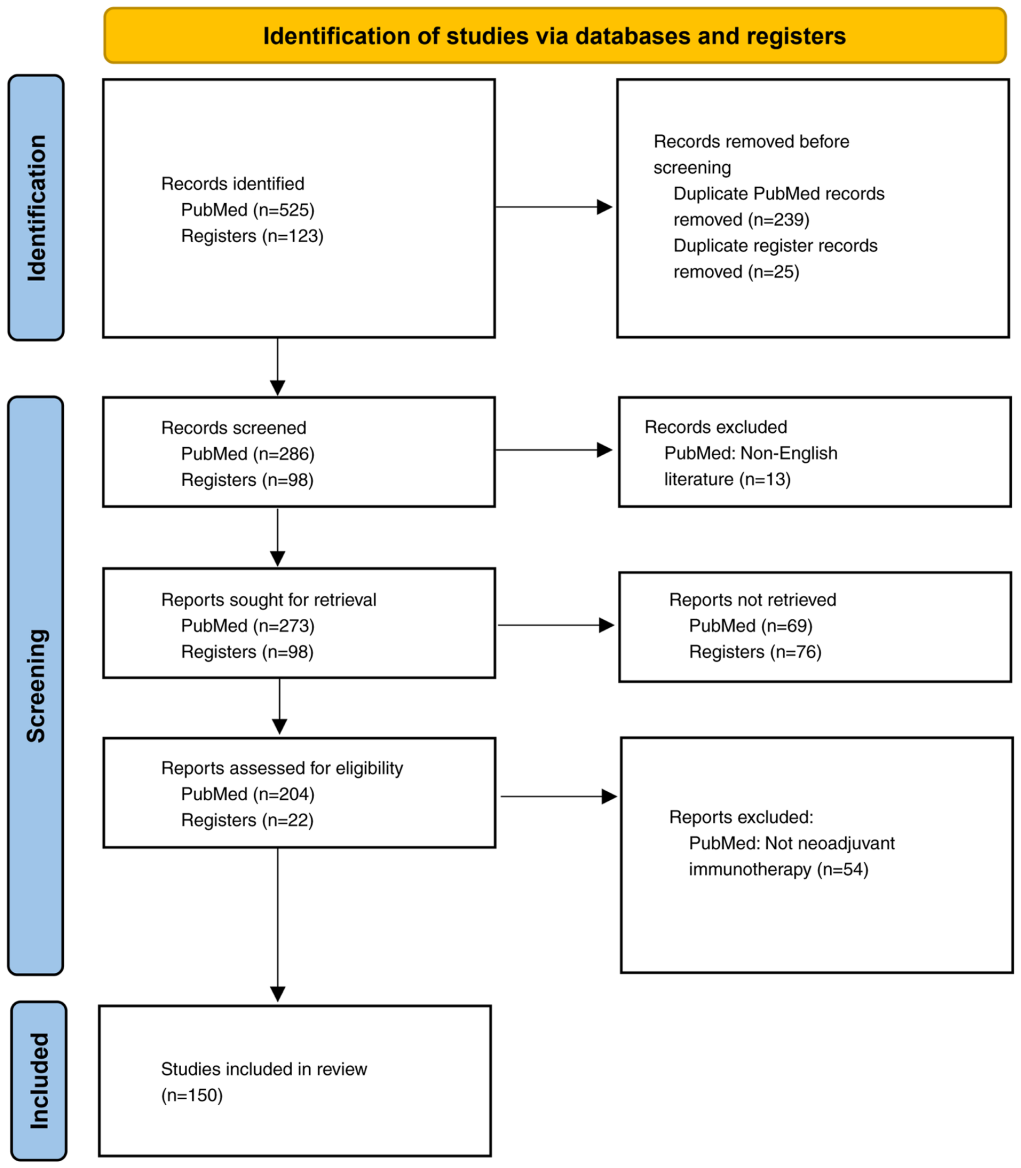
Preferred Reported Items for Systematic Reviews and Meta-Analyses 2020 flow diagram for new systematic reviews that include searches of databases and registries only.

The present study was reported in accordance with the Preferred Reported Items for Systematic Reviews and Meta-Analyses guidelines for systematic reviews (56). Articles were sourced from PubMed (pubmed.ncbi.nlm.nih.gov/) and registered clinical study were sourced from clinicaltrials.gov (clinicaltrials.gov/) using the search terms ‘neoadjuvant immunotherapy’, ‘radiofrequency ablation’, ‘liver transplantation’, ‘hepatocellular carcinoma’, ‘liver cancer’, ‘intrahepatic cholangiocarcinoma’ and ‘HCC’, covering the period from January 1950 to March 2025. The review was limited to papers published in English. A total of 526 studies was retrieved. After screening the studies, 150 were reviewed in detail (Fig. 1) for their originality and relevance to the broader scope of the present review.

## 3. Clinical and scientific potential of neoadjuvant immunotherapy for HCC

Neoadjuvant therapy is used to kill cancer nest cells or small lesions, decrease the tumor size before surgery, decrease postoperative recurrence and improve the postoperative survival rate. In general, neoadjuvant therapy serves two purposes: To decrease the likelihood of recurrence due to the multicentric origin of other lesions that are not detectable by imaging and to shrink the primary tumor cells to facilitate the final treatment. The achievements of immunotherapy in managing advanced HCC (20,29) and the positive outcomes of perioperative immunotherapy for other malignant tumors have resulted in FDA approval (48,57–60). Thus, ICIs may be utilized as neoadjuvant therapy of resectable HCC (26). PD-1/PD-L1 antibody neoadjuvant therapy has gained attention, with previous studies supporting its efficacy (35,54,61). Such drugs have the potential to increase the RFS rate without markedly delaying surgery (51,54,62–64). Neoadjuvant immunotherapy has been demonstrated to confer more surgical opportunities to patients with cancer with unresectable lesions (65,66). Ongoing clinical trials are examining the role of perioperative immunotherapy in treating resectable HCC and ICC (Tables I and II).

## 4. Neoadjuvant immunotherapy before liver transplantation (LT)

The 5-year survival rate for patients with HCC is between 17 and 55% (67). The recurrence rate following LT reaches 10%, even among patients who meet Milan's criteria (Single tumor diameter ≤5 cm, or up to 3 tumors with each diameter ≤3 cm, without vascular invasion or extrahepatic metastasis) (68) and LT is limited to eligible patients with cancer and cirrhosis (69). LT does not ensure lifelong disease control in a certain proportion of patients with HCC, which indicates that combined treatment require research.

Neoadjuvant systemic therapy is under evaluation in transplant oncology as a strategy to improve outcomes for patients with HCC. Preoperative intratumoral neoadjuvant immunotherapy, which includes IL-12 gene and dendritic cell therapy, effectively controls tumor recurrence in patients with HCC post-LT and may be used in more cancer types (70). Neoadjuvant immunotherapy, including nivolumab (71) or atezolizumab and bevacizumab (72), has been used to downstage HCC in patients who exceed the Milan criteria before LT. Pembrolizumab also shows potential for downstaging, as evidenced by a 16.2% partial response rate in the KEYNOTE-240 trial (73); however, its use immediately before transplantation leads to a higher risk of organ rejection. Although the use of ICIs immediately before or after transplantation may lead to mortality (74), a prospective database includes guidelines on how and when to administer ICIs to transplant recipients (75). A multicenter evaluation of patients with HCC receiving ICIs pre-LT demonstrated favorable survival and safety outcomes (76). Numerous approved ICIs (nivolumab, atezolizumab) (76) have been studied as neoadjuvant therapies for LT recipients with HCC, and the results have shown a low rejection rate when a washout period (interval between stopping immunotherapy before LT) is implemented prior to LT (77).

Neoadjuvant treatment can increase the number of transplantation opportunities for patients awaiting LT and aid identification of patients who will benefit most from this approach. Patients who were previously unable to undergo surgery may be downstaged through neoadjuvant therapy, which may provide a chance for LT and enable screening for the effectiveness of systemic therapy. Benefits of this strategy have been demonstrated for patients with HCC in a previous randomized controlled trial (78). Integrating ICIs with local regional therapy, or starting ICIs during such treatment, may slow disease progression and improve LT outcomes. Neoadjuvant PD-1-targeted immunotherapy combined with TKIs shows promising efficacy and a low mortality rate in transplant recipients under clinical supervision (53).

A retrospective study was performed to analyze 159 patients with HCC who received LT, of whom 39 (24.5%) received pretransplant ICI treatment. The acute rejection and rejection-associated mortality rates during the perioperative period in the ICI group were 23.1 (9/39) and 12.8% (5/39), respectively, which were markedly higher compared with those in the non-ICI group [5 (6/120 vs. 0% (0/120;)], but the administration of pretransplant ICI treatment improved the survival outcomes of patients with microvascular invasion-positive HCC following transplantation (79). A retrospective analysis revealed that 25 patients with HCC received ICIs before LT, and PD-1 was the most commonly used ICI (68%; 17/25). The median interval between the last dose of ICI and LT was 64 days (range, 40.00–150.75). The authors also performed a literature review, in which a total of 96 patients with HCC who had received ICIs prior to LT were included. The PD-1 inhibitor monotherapy group had a notably higher rejection rate compared with the groups receiving PD-L1 inhibitor monotherapy and other ICI combination regimens. In patients receiving pembrolizumab, the interval from ICI therapy to LT was shorter in the rejection compared with in the non-rejection group (80). A total of 21 patients (21.88%) experienced transplant rejection, and three patients (14.29%) died following transplant rejection (80).

A systematic review and meta-analysis of data from 91 patients with HCC and patients who received ICIs prior to LT identified 24 (26.4%) cases of allogeneic rejection (81). A 3-month washout period may decrease this risk to the level of that in patients who have not been exposed to ICIs. In addition, longer immunotherapy cycles and a tumor burden within the Milan criteria may indicate a decreased risk of HCC recurrence. However, this observation needs to be validated in larger prospective studies (81). The use of neoadjuvant atezolizumab and bevacizumab before LT in patients with HCC is safe and effective, with an objective response rate (ORR) of 94% (CR, 59%), parameters meeting the Milan criteria (82%) and a pathological response rate of 88%. Grade 3–4 treatment-related adverse events occurred in 17.6% of the cases and were controllable. The 1- and 3-year survival rates following LT were 94.2 and 88.2%, respectively (82). A multicenter evaluation of ICIs and local treatment prior to LT revealed good survival and safety outcomes with no grade 4–5 adverse events (76). A total of seven patients (5.98%) experienced rejection after LT, with six patients (5.13%) receiving the final dose of ICI <3 months prior to LT (76). For patients with HCC receiving atezolizumab, nivolumab or pembrolizumab ICI treatment, the 42-day period before LT is the safest washout period (83); however, researchers hypothesize that the washout period should be >3 months because the rejection rate is lower (81). The aforementioned studies indicate that neoadjuvant immunotherapy for LT has a good pathological response and safety and can improve patient prognosis. However, prospective studies and biomarkers are needed to determine the safety and efficacy of immunotherapy regimens before and after LT. Associated clinical trials are still in progress (trial nos. NCT04425226 and NCT05027425).

Although the prospect of immunotherapy is notable, ICI therapy should be avoided in patients with recurrence following LT because of the high rejection rate of allograft transplantation (84). Furthermore, the intricate nature of HCC necessitates a cautious approach to the use of ICIs in transplant candidates. A clinical concern associated with ICI treatment is hepatotoxicity, which is a notable issue that must be considered for patients with limited liver function. Presently, patients at high risk of recurrence following LT, particularly those with multifocal tumors, elevated a-fetoprotein levels and notable tumor volumes, are considered optimal candidates for neoadjuvant ICIs (85). Other strategies are needed to identify biomarkers to improve patient eligibility. Advancements in transplantation oncology, particularly in local and systemic therapies such as immunotherapy, have expanded options for exploring neoadjuvant and adjuvant strategies to increase HCC resection rates (86,87).

## 5. Neoadjuvant immunotherapy before liver resection

Elias *et al* (88) investigated the effects of prehepatectomy immunostimulation via recombinant IL-2 (rIL-2) and evaluated its tolerance for extensive hepatectomy. The aforementioned study revealed that the toxicity experienced during rIL-2 infusion was manageable, suggesting that the infusion of rIL-2 before extensive hepatectomy for liver metastasis originating from colorectal cancer (CRC) is well tolerated and can reverse postoperative immunosuppression. This is equivalent to the predecessor of immunotherapy. A vaccine composed of 2.0 each heat shock protein 70- and glypican 3-derived peptide, 1.4 poly-ICLC and 1.0 mg hLAG-3Ig is safe for perioperative immunotherapy in patients with human leukocyte antigen-matched HCC and effectively promotes CD8^+^ T cell tumor infiltration (89).

Phase 1b trial results reveal that the neoadjuvant nivolumab/ipilimumab combination may improve survival outcomes in patients with HCC (55). Kaseb *et al* (35) performed a phase 2, single-center, open-label, randomized trial to assess the impact of perioperative immunotherapy on potentially resectable HCC. In the midterm analysis, the trial achieved its primary goal of safe treatment and no delay in surgical resection and reported a 25% pathological complete response (pCR) rate. Post-trial, group A comprised 13 patients who underwent three cycles of neoadjuvant nivolumab, whereas group B included 14 patients who received a combination of neoadjuvant nivolumab and ipilimumab. A major pathological response (MPR) was observed in 33% of patients treated with nivolumab monotherapy, whereas 27% of those who received nivolumab and ipilimumab experienced a response. The use of perioperative nivolumab, either alone or in combination with ipilimumab, is safe and feasible for treating patients with resectable HCC (35).

High levels of cytotoxic effector CD8^+^ T cell infiltration in tumors following perioperative immunotherapy are associated with a pCR (90). A patient with metastatic Merkel cell carcinoma (MCC) and biopsy-confirmed moderately differentiated HCC was treated with avelumab (10 mg/kg every 2 weeks for 15 cycles), which led to complete remission of MCC. Likewise, the arterial enhancement in the HCC lesion vanished entirely, indicating that the lesion had undergone necrosis (91). Pretreatment biopsy immunohistochemistry revealed negative PD-L1 expression on tumor cells, and tumor-infiltrating lymphocytes (TILs) were predominantly CD3^+^ and CD8^+^. Post-treatment analysis of resected samples revealed a 2-fold increase in CD3^+^ and CD8^+^ T cell infiltration and a 1.5-fold increase in forkhead box P3 (FoxP3) expression (91). These findings align with those of Kaseb *et al* (90) on pCR following perioperative ICI combination therapy. Furthermore, the aforementioned study discovered a highly cytotoxic CD8^+^ T cell subset specific to tumors within TILs. This demonstrates that preoperative immunotherapy is effective for enhancing specific antitumor immunity.

A phase 2, single-arm, open-label trial assessed the impact of two cycles of neoadjuvant cemiplimab on patients with resectable stage Ib, II and IIIb HCC. Of 21 patients, 20 (95%) underwent successful resection; four (20%) had notable tumor necrosis and 3 (15%) had a partial response. The remaining patients exhibited stable disease. To the best of our knowledge, the aforementioned report is the most extensive clinical trial to date on neoadjuvant anti-PD-1 monotherapy for HCC (54).

Pembrolizumab, a PD-1 inhibitor, is being studied as a perioperative therapy before curative treatments such as hepatic resection or radiofrequency ablation (73). A phase 2, single-arm trial (trial no. NCT05471674) evaluating the clinical benefit of nivolumab in patients with untreated, borderline resectable HCC has completed enrollment, with results yet to be reported. While phase 3 randomized clinical trials are lacking, phase 2 studies have demonstrated promising interim results regarding the efficacy of neoadjuvant ICI treatment for HCC (35,54).

Neoadjuvant immunotherapy has become a standard treatment for numerous tumor types (breast and lung cancer *et al*) because of its association with improved survival outcomes. However, HCC presents a high level of heterogeneity, so it is different from other malignant tumors. The impact of clinical stage on the outcome of neoadjuvant immunotherapy for patients with HCC is notable, as heterogeneity increases with tumor volume. Furthermore, ICIs possess satisfactory toxicity traits. Hence, further research is needed to assess the potential therapeutic advantages of preoperative PD-1 blockade in individuals with resectable HCC. The identification of markers to predict immunotherapy efficacy is key for improving neoadjuvant treatment outcomes. Improved predictive markers of treatment response that can be assessed using solid and liquid biopsy are also key for the incorporation of immunotherapy into HCC management (92).

A total of nine studies of neoadjuvant ICIs for resectable HCC were included in a previous meta-analysis, which revealed a pCR rate of 12.9% (95% CI, 6.7–19.1%) and an MPR rate of 27.3% (95% CI, 15.1–39.4%) (31). Subgroup analysis failed to demonstrate the superiority of any individual ICI or combination therapy. The aforementioned study revealed that neoadjuvant ICIs are well tolerated by patients with resectable HCC and have therapeutic benefits, based on histopathological response results. Another meta-analysis included 11 studies; the overall MPR rate of neoadjuvant immunotherapy for patients with resectable HCC was 0.47 (95% CI, 0.31–0.70), and the pCR rate was 0.22 (95% CI, 0.14–0.36). The overall ORR was 0.37 (95% CI, 0.20–0.69) (93). There was no notable difference in the efficacy and safety of monotherapy immunotherapies. The efficacy of dual ICI combination therapy is superior compared with targeted combination and single immunotherapy. A cross-trial analysis of the pathological response data of 104 patients who used ICIs [predominantly ICI combinations (69%)] before resection revealed that 33 patients (32%) had an MPR and 19 (18%) had a CR (94). The RFS of patients with an MPR was markedly longer compared with that of patients without an MPR. A tumor regression rate of 90% was used as the optimal threshold for predicting RFS. Neoadjuvant immunotherapy is safe and feasible for treating patients with resectable HCC.

## 6. Locally advanced HCC (according to the Barcelona Clinic Liver Cancer system)

To the best of our knowledge, no studies have reported neoadjuvant immune monotherapy for advanced HCC. On the other hand, neoadjuvant therapy for advanced HCC involves combination therapy, such as immunotherapy combined with targeted therapy. Chen *et al* (95) presented a case report showing that combined treatment with lenvatinib and nivolumab is effective and safe and could enable patients with extensive HCC to receive extended right hepatectomy. In patients with locally advanced HCC treated with nivolumab and cabozantinib as neoadjuvant therapy, the margin-negative resection rate was 80% and the MPR rate was 42% (61). A clinical trial (trial no. NCT03299946) evaluated the efficacy of neoadjuvant cabozantinib combined with nivolumab followed by definitive resection in patients with locally advanced HCC. The aforementioned trial was a single-arm and open-label phase 1b study (96). Among the 15 enrolled patients, 12 (80%) had successful margin-negative resections, with five of these patients (42%) achieving an MPR (61). Patients who received neoadjuvant immunotherapy had margin-negative resection and RFS rates similar to those of patients who underwent upfront surgical resection in a clinical trial (trial no. NCT03299946) (97).

Administering immunotherapy at an early stage may improve the outcomes of patients with resectable HCC (38). The increasing detection rate of small HCC lesions facilitates the assessment of the necessity and efficacy of neoadjuvant immunotherapy for these patients prior to resection, radiofrequency ablation or LT (93,98). This includes evaluating the efficacy, side effect-to-efficacy ratio and cost-effectiveness. Neoadjuvant immunotherapy selection for patients with HCC is performed according to patient features, such as medication cycle and surgery eligibility and requires perspectives from both hepatobiliary surgeons and immunologists (87).

As the recurrence rate of HCC is still high after resection, there remains a need to increase disease control through neoadjuvant methods. Clinical guidelines also support research in the field of neoadjuvant therapy (99). Neoadjuvant immunotherapy improves tumor control more effectively compared with adjuvant immunotherapy by boosting and maintaining the tumor-specific immune response (50). Neoadjuvant immunotherapy may signify a paradigm shift in the treatment of resectable HCC. The traditional Response Evaluation Criteria in Solid Tumors (RECIST) 1.1 standard underestimates the previously reported response of melanoma to immunotherapy (100). Prolonged exposure to PD-L1 inhibitors is associated with a pCR and complete disappearance of intratumoral arterial enhancement, while the tumor size does not decrease (91). This indicates a complete radiological response [according to the modified (m)RECIST for liver cancer] or stable disease (according to the RECIST 1.1) (101). Consequently, selecting the appropriate criteria (mRECIST, immune RECIST or RECIST 1.1) for assessing the response of patients with HCC to immunotherapy is important for future research (102).

A previous study involved 20 treatment-naive patients with HCC with intermediate and locally advanced tumors who received preoperative nivolumab (3 mg/kg for three cycles) before surgical resection (103). Among the 19 patients who underwent surgical resection, seven (36.8%) exhibited major pathological tumor necrosis (≥60%), including three with nearly complete (>90%) necrosis. No patients experienced notable adverse reactions that contraindicated hepatectomy. RNA-sequencing analysis of tumor biopsy samples before nivolumab treatment and resected samples following treatment revealed a marked increase in CD8^+^ T cells in the HCC tissue of patients exhibiting major pathological necrosis. The aforementioned study also identified a useful non-invasive biomarker (the copy number variation-based anti-PD-1 score) for predicting responsiveness. The aforementioned study indicated that neoadjuvant nivolumab demonstrates promising clinical efficacy in patients with intermediate and locally advanced HCC. In addition, a retrospective study analyzed 36 patients with HCC at high risk of recurrence who received neoadjuvant immunotherapy (97). The negative margin resection and RFS rates were similar with those of patients who underwent one-stage surgical resection. Neoadjuvant immunotherapy may enable high-risk patients, including those who do not meet the resection criteria, to undergo negative resection at the margin and achieve long-term clinical outcomes comparable with those of one-stage resection.

## 7. Neoadjuvant immunotherapy before radiofrequency ablation

Radiofrequency ablation kills tumor cells through high-temperature coagulation necrosis and is minimally invasive with a fast recovery, low cost, and definite therapeutic effect. At present, the indication for radiofrequency ablation treatment of HCC is a lesion with a diameter of ≤3 cm (104). However, the indications are expanding, and numerous larger tumors can be treated with radiofrequency ablation (105). These patients can receive adjuvant immunotherapy following radiofrequency ablation treatment (106). In addition, radiofrequency ablation is often used for patients who experience recurrence following resection surgery (107). Therefore, neoadjuvant immunotherapy may be suitable for patients undergoing radiofrequency ablation. To the best of our knowledge, however, there are no relevant reports.

## 8. Electroporation

Electroporation is a technique that uses electrical pulses to form temporary micropores on the cell membrane. When high-intensity electrical pulses are applied, a potential difference is formed on both sides of the cell membrane, leading to polarization of the phospholipid bilayer (108,109). It is divided into reversible and irreversible electroporation. Irreversible electroporation is achieved by applying high voltage pulses through electrode needles. Under high electric field strength, the pores cannot be repaired, leading to cell apoptosis or necrosis, selectively killing tumor cells. It is a non-thermal mechanism that avoids damage to surrounding tissues caused by thermal ablation (110). Electroporation induces T cell recruitment, which may promote immunomodulation (111). A phase 2 clinical trial (trial no. NCT03630640) aims to evaluate the use of nivolumab with neoadjuvant electroporation for curative purposes.

## 9. Neoadjuvant immunotherapy for ICC

Neoadjuvant chemotherapy and radiotherapy are to treat extrahepatic cholangiocarcinoma, including distal and perihilar types (112). To the best of our knowledge, there are few case reports on ICC neoadjuvant immunotherapy (113,114). Dual immunotherapy combined with chemotherapy may also be feasible. A previous study have shown that the combination of a gemcitabine and cisplatin (GC) regimen with durvalumab and tremelimumab as first-line treatments for advanced biliary tract cancer (BTC) has an ORR of up to 70% (115). Therefore, a clinical study is underway to evaluate the use of a GC regimen combined with durvalumab and tremelimumab as a neoadjuvant therapy strategy for BTC (trial no. NCT06017297). Most registered clinical trials involve immunotherapy combined with chemotherapy and other treatment (Table II). In the field of neoadjuvant therapy, combinations of multiple chemotherapy drugs are more common compared with immunotherapy and chemotherapy combinations, because chemotherapy is the primary treatment for cholangiocarcinoma, but immunotherapy may improve the efficacy of these treatments.

## 10. Other concerns regarding neoadjuvant immunotherapy

The standard clinical practice for treating early-stage HCC involves surgical resection. However, HCC has high recurrence rates following surgery. Clinical research is needed to determine whether preoperative treatment enhances survival and decreases recurrence in patients with China Liver Cancer (CNLC) (116) stage Ia, Ib and IIa cancer. Neoadjuvant therapy is used for patients with resectable mid- to late-stage HCC (CNLC stage IIb, IIIa) to improve oncological characteristics, with the aim of decreasing postoperative recurrence and extending survival.

Neoadjuvant immunotherapy elicits pathological responses and may decrease the risk of postoperative recurrence in patients with HCC. The recurrence of HCC may differ from that of other types of carcinomas because it often occurs in a metachronous multicentric manner. Immunotherapy capable of eliciting systemic and long-lasting responses may serve as an attractive treatment option. Clinical studies on ICIs for HCC treatment have shown promising results, despite the suppressive environment of the liver and tumor immune evasion mechanisms (35,54). Short-term recurrence may occur in a metachronous multicentric manner, and these patients may benefit from neoadjuvant therapy. Hence, certain studies have focused on assessing the safety and tolerance of perioperative immunotherapy in individuals with resectable HCC (35,54). To the best of our knowledge, there is no published research on whether neoadjuvant immunotherapy prolongs survival. The evidence suggests that immunotherapy, whether as monotherapy or in combination, can be used preoperatively or perioperatively for patients with resectable HCC to improve surgical outcomes (35,54).

A previous study demonstrated that the neoadjuvant combination of tremelimumab and durvalumab administered prior to CRC liver resection is safe (117). However, the efficacy of neoadjuvant immunotherapy in the treatment of ICC is unclear. A case report described a patient with stage IIIb ICC with lymph node metastasis who was successfully treated via neoadjuvant therapy including camrelizumab and cisplatin (113). The selection of neoadjuvant immunotherapy drugs for ICC should be based on the first-line immunotherapy regimen for advanced BTC, with durvalumab or pembrolizumab (which have been approved by the US Food and Drug Administration) for the treatment of advanced BTC) as the preferred options (118,119). In addition, the Chinese Society of Clinical Oncology guidelines (120) suggest that the use of toripalimab (121) and camrelizumab (122,123) is effective and safe in the treatment of advanced BTC on the basis of phase 2 trials. At present, the ORR of PD-1/PD-L1 inhibitors combined with a GC regimen for the treatment of advanced BTC is 25–30% (124,125). Other PD-1/PD-L1 inhibitors that have not yet been studied but have similar mechanisms to those of the aforementioned antibodies may be effective and safe in treating other solid tumors (breast and lung cancer).

According to the mechanism and characteristics of immunotherapy, whether the cancer exhibits pseudoprogression needs to be confirmed when immunotherapy is applied. Pseudoprogression is tumor growth caused by therapeutic effects but not disease progression. This may entail a short-lived increase in tumor size and the emergence of new lesions without any decline in the condition of the patient. Following pseudoprogression, individuals may present with stable disease or tumor regression. Pseudoprogression may occur within or beyond 12 weeks of initial treatment, potentially due to inflammatory cell infiltration into tumor tissue or edema and necrosis caused by immunotherapy (126,127). After confirming progression, the decision of whether to maintain immunotherapy is based multiple factors, such as the pretreatment regimen, drug resistance and disease progression mode, patient factors and immunotherapy safety (128).

A notable challenge associated with neoadjuvant immunotherapy is the potential for treatment delay or discontinuation due to disease progression or toxicity associated with the therapy. Future research should involve further characterization of treatment response and predictive factors, optimization of drug combinations and treatment durations in the neoadjuvant setting, and assessment of the necessity of follow-up adjuvant treatment for all patients. To the best of our knowledge, there is no recognized biomarker that can predict the resistance or response of patients with HCC to immunotherapy. In addition, biomarkers established in other types of cancer, such as PD-L1 expression and tumor mutation burden, do not have stable predictive effects in HCC (129). Therefore, whether PD-L1 immunohistochemistry can guide immunotherapy decision making for HCC remains controversial. More research is needed to identify dependable biomarkers for predicting therapeutic response. Ultimately, selecting suitable patients remains crucial, as unsuccessful immunotherapy can result in disease progression, thereby limiting treatment options. Neoadjuvant immunotherapy is a promising option for perioperative treatment (98). Extensive clinical trials of this approach are needed to justify its clinical application.

The immunotherapy response and immune tolerance involve complex mechanisms. Firstly, immune suppressive cells in the tumor microenvironment, such as regulatory T cells (Tregs), highly express FoxP3, CTLA-4 and CD25, which suppress effector T cell function by secreting factors such as IL-10 and TGF-β (130). Myeloid derived suppressor cells (MDSCs) serve a key role in immune suppression and tumor immune evasion through direct and indirect mechanisms. MDSCs not only secrete immunosuppressive factors such as IL-10, TGF-β, reactive oxygen species and prostaglandin E2, but also enhance immunosuppressive effects by promoting the expansion of Tregs (131–133). In addition, MDSCs promote tumor immune evasion and increase cell invasion capacity by upregulating PD-L1 expression and promoting epithelial to mesenchymal transition (134). NK cell deficiency promotes immune escape (135). Secondly, inhibitory cytokines, such as TGF-β, inhibit T cell and NK cell activity, promoting Treg differentiation (136).

ICIs may cause reactivation of hepatitis B virus (HBV). Research indicates that ICIs are safe and effective for patients with advanced cancer and HBV infection (137). Clinical monitoring of liver enzymes and HBV DNA is necessary during ICI therapy (137). Further prospective studies are needed to assess the risk of HBV reactivation in patients receiving ICI treatment (138). There is controversy over whether ICIs are a high risk factor for the reactivation of chronic HBV or are effective in the treatment of HBV (139,140). ICIs are safe for patients with solid tumors and hepatitis C virus infection (141). ICIs may be less effective in patients with non-viral HCC, especially those with non-alcoholic fatty liver disease (142).

To the best of our knowledge, research on the safety of ICIs before LT is limited to small sample study (80,143). There is risk of transplant rejection when ICIs are used before LT. The risk of rejection after LT in patients with HCC with pretransplantation ICI treatment is not yet fully understood. It is also unclear how long it takes to perform LT after ICIs are discontinued and the safe time window between administration and LT. Some studies suggest that the use of ICIs before LT and associated factors, particularly the type of ICI and the interval between ICI therapy and LT, are associated with the risk of transplant rejection (80,143). Multicenter prospective studies are needed to explore the safety of ICIs.

Common side effects of immunotherapy include immune-associated lung and liver injury and thyroid dysfunction, skin gastrointestinal toxicity and endocrine system abnormality. The risk of immune-mediated hepatitis (IMH) is unpredictable, and this condition is often accompanied by positive autoantibodies, elevated immunoglobulin levels or lymphocyte infiltration, regardless of the dosage and course of treatment (144). The incidence of acute hepatitis caused by immunotherapy for metastatic cancer is 3.5% (145). The primary mechanism by which ICIs induce IMH is the overactivation of T cells. The reported incidence rate of IMH is between 1 and 15% (146) . IMH presents histologically as whole lobular hepatitis (~70%), isolated central area necrosis (~20%), primarily granulomatous hepatitis (~5%) and other mild forms of tissue damage (~5%) (147). Therefore, early monitoring of liver function is necessary. For mild liver injury, immunotherapy may be suspended and patients should be closely monitored. If liver injury is severe, shock therapy with glucocorticoids may be necessary. Clinicians need to educate and self-manage patients and collaborate across multiple disciplines to develop personalized plans (148).

Neoadjuvant immunotherapy for liver cancer has been applied in different clinical scenarios (LT and liver resection) and liver tumors (HCC and ICC) (35,71,72,115). In the surgical resection of patients with HCC, owing to the early stage of the disease, it is possible to use only neoadjuvant immunotherapy (35); however, for the LT population, combination therapy is often used as neoadjuvant therapy (53). For ICC, combination therapy is commonly used as neoadjuvant therapy (115). Currently, immunotherapy is more commonly used for advanced liver cancer. The use of neoadjuvant therapy for HCC, particularly the use of immunosuppressants as a single drug for neoadjuvant therapy, is not as well supported and common as for other tumors (149); it is more commonly used for tumor downgrading. In the analysis of the characteristics of the patients with HCC receiving neoadjuvant therapy included in current clinical studies, the population receiving preoperative neoadjuvant ICI immunotherapy generally met the criteria for one-stage resection. Therefore, postoperative adjuvant therapy may be considered equivalent in efficacy to neoadjuvant therapy; notably, adjuvant therapy can prevent possible complications caused by neoadjuvant therapy, such as immune hyperprogression, which can lead to missed surgical opportunities. Future research should focus on whether patients with resectable or small liver cancer benefit from neoadjuvant immunotherapy (150). Studies should also aim to define the criteria for population screening and treatment efficacy evaluation.

Immunotherapy is increasingly applied as neoadjuvant therapy for liver cancer to improve surgical outcomes. Joint efforts of surgeons and immunologists are needed to determine how to expand the population of patients who may benefit from neoadjuvant immunotherapy.

## Figures and Tables

**Table I.** Clinical trials of neoadjuvant immune checkpoint inhibitors for HCC.

Treatment regimen (status)	NCT no.	Study design	Notable inclusion criteria	Number of patients	Treatment cycle, intervention	Study outcomes (primary end point)	(Refs.)
Ipilimumab +	NCT03682276	Phase 1/2,	Resectable HCC	32	Nivolumab, 3 mg/kg, Q3W ×2 +	Delay of surgery (up to day 89);	(74)
nivolumab		single-center,			ipilimumab, 1 mg/kg, Q3W ×1; OP	incidence of treatment-emergent	
(recruiting)		single-arm,			on day 43	adverse events (up to day 127)	
		open-label					
Nivolumab with	NCT03222076	Phase 2,	Resectable HCC	30 (final	Arm A: Nivolumab, Q2W ×3; liver	Incidence of adverse events	(55)
or without		single-center,		enrollment,	surgery (day 1 of week 7); nivolumab	(5 years)	
ipilimumab		randomized,		27)	Q4W to 2 years. Arm B: ipilimumab,		
(completed)		open-label			day 1 + nivolumab, Q2W ×3; liver		
					surgery (day 1 of week 7); nivolumab		
					Q4W ± ipilimumab Q6W to 2 years		
Nivolumab +	NCT03510871	Phase 2,	Potentially	40	Nivolumab, 3 mg/kg + ipilimumab,	Proportion of subjects with	
ipilimumab		multicenter,	resectable HCC		1 mg/kg, Q3W ×4; OP	tumor shrinkage (>10%	
(recruiting)		single-arm,				decrease in tumor size;	
		open-label,					
Nivolumab	NCT05471674	Phase 2,	Untreated,	20	Nivolumab, 3 mg/kg, Q2W ×3; OP	Pathological tumor response rate	
(completed)		single-center,	borderline		at 2 weeks after the 3rd dose		
		single-arm	resectable HCC				
Nivolumab (active,	NCT03630640	Phase 2,	Advanced HCC	43	Nivolumab, 240 mg, Q2W ×2; EP;	Local RFS for 1 year	
not recruiting)		multicenter,	treated by		480 mg Q4W ×13		
		single-arm	electroporation				
Nivolumab +	NCT03812562	Phase 1,	Resectable HCC,	2	Yttrium Y90, within 1–2 weeks start;	3-year recurrence rate	
yttrium Y90 glass		single-center,	Child-Pugh		nivolumab, Q2W ×4; OP		
microspheres		single-arm	score ≤8				
Pembrolizumab	NCT03337841	Phase 2,	Resectable or	50	Pembrolizumab, 200 mg ×1; resection	1-year RFS rate	
(not yet recruiting)		single-arm	RFA of HCC		or RFA; 200 mg, Q3W ×16		
Pembrolizumab	NCT04224480	Phase 1,	Resectable HCC,	45	Pembrolizumab, 200 mg ×1; OP;	Number of subjects with HCC	
(recruiting)		single-center,	Child-Pugh		200 mg, Q3W ×13	recurrence (2 years); number of	
		single-arm	score ≤6			CD8^+^ Ki67+ T cells in resected	
						tumors from subjects	
Cemiplimab	NCT03916627	Phase 2	Resectable HCC	21	Cemiplimab 350 mg, 2 doses; surgery;	Notable tumor necrosis at time	(54)
(completed)					8 doses	of surgery	
Toripalimab	NCT03867370	Phase1/2,	Resectable HCC	40	Arm A: Toripalimab, 480 mg ×1; OP;	Pathological response rate	
with or without		randomized,			240 mg, Q3W ×16. Arm B: Toripalimab,		
lenvatinib		open-label,			480 mg ×1 + lenvatinib, 8 or 12 mg		
(terminated)		multicenter			qd; OP; 240 mg, Q3W + lenvatinib		
					up to 48 weeks. Arm C: Toripalimab,		
					480 mg ×1 + lenvatinib, 8 or 12 mg; qd;		
					OP; 240 mg, Q3W ×16		
Tislelizumab with	NCT04615143	Phase 2, non-	Resectable	80	Arm A: Tislelizumab, 200 mg, Q3W ×2;	1-year disease-free survival	
or without		randomized,	recurrent HCC		OP; 200 mg Q3W for 1 year. Arm B:		
lenvatinib		single-center			Tislelizumab, 200 mg, Q3W ×2 +		
(recruiting)					lenvatinib, 8/12 mg qd ×4W; OP;		
					tislelizumab, 200 mg, Q3W + lenvatinib,		
					8/12 mg qd for 1 year		
Tremelimumab +	NCT02754856	Phase 1,	Colorectal cancer	22 (final	Tremelimumab, 75 mg ×1 +	Feasibility (number of patients	(81)
durvalumab		single-center,	with resectable	enrollment,	durvalumab, 1,500 mg ×1; OP;	who successfully undergo sur-	
(active, not		single-arm,	liver metastases	24)	durvalumab, 1,500 mg Q4W ×4	gery, up to 17 weeks). Incidence	
recruiting)		open-label				of adverse events (1.5 years)	
Nivolumab with or	NCT04658147	Phase 1,	Potentially	20	Arm A: Nivolumab, 480 mg, Q4W ×2;	Number of patients who	
without relatlimab		single-center,	resectable HCC		OP; 480 mg Q4W for 1 year. Arm B:	complete preoperative	
(recruiting)		randomized,			Nivolumab, 200 mg, Q4W ×2 +	treatment and proceed to	
		open-label			relatlimab, 480 mg, Q4W ×2; OP;	surgery	
					nivolumab, 200 mg, Q4W + relatlimab,		
					480 mg, Q4W for 1 year		
Pembrolizumab	NCT05185739	Phase 2,	Resectable HCC	60	Arm A: Pembrolizumab, 200 mg,	MPR rate	
and lenvatinib		randomized,			Q3W ×2; OP; pembrolizumab, 200 mg,		
(recruiting)		multicenter			Q3W for 1 year. Arm B: Lenvatinib,		
					8/12 mg qd ×6W; OP; lenvatinib,		
					8/12 mg qd for 1 year. Arm C:		
					Pembrolizumab, 200 mg, Q3W ×2 +		
					lenvatinib, 8/12 mg qd ×6W; OP;		
					pembrolizumab+ lenvatinib for 1 year		
Durvalumab and	NCT05440864	Phase 2,	Resectable HCC	28	Tremelimumab, 300 mg ×1 +	Number of grade >3 adverse	
tremelimumab		open label,			durvalumab, 1,500 mg, Q4W ×2; OP;	events	
(recruiting)		single-arm,			durvalumab, 1,500 mg, Q4W ×11		
		multicenter					
Durvalumab and	NCT05027425	Phase 2,	HCC (patients	30	Durvalumab, 1,500 mg IV, Q4W+	Cellular rejection rates	
tremelimumab		single-arm,	listed for an LT),		tremelimumab 300 mg IV, 1 dose	(up to 30 days post LT)	
(active, not		open-label,	HCC within		on day 1 of only the first cycle for		
recruiting)		multicenter	UCSF criteria,		up to 4 months; locoregional		
			Child-Pugh score		therapy; LT		
			≤7 and ECOG PS				
			of 0 or 1				

OP, operation; W, week; QD, once a day; RFS, recurrence-free survival; MPR, major pathological response; EP, electroporation; LT, liver transplantation; IV, intravenous; HCC, hepatocellular carcinoma; NCT, national clinical trial; RFA, radiofrequency ablation; UCSF, University of California San Francisco; ECOG PS, Eastern Cooperative Oncology Group performance status.

**Table II.** Clinical trials of neoadjuvant immune checkpoint inhibitors for ICC.

Treatment regimen	NCT no.	Study design	Notable inclusion criteria	Number of patients	Treatment cycle, intervention	Study outcomes (primary end point)
Durvalumab +	NCT05672537	Phase 2, single-center,	Resectable ICC	70	Arm A: Durvalumab, 1,500 mg, Q3W, +	1-year relapse-free
gemcitabine +		single-arm, randomized,			gemcitabine, 1,000 mg/m^2^, D1 and D8,	survival rate
cisplatin		open-label			Q3W + cisplatin 25 mg/m^2^, D1 and D8,	
					Q3W. Arm B: Surgery	
Tislelizumab +	NCT05557578	Phase 2, single-center,	Resectable ICC	20	Tislelizumab, 200 mg, day 1, Q3W ×6 +	Objective response
GEMOX		single-arm, prospective,			gemcitabine 1,000 mg/m^2^, D1 and D8,	and R0 resection rate
		exploratory, open-label			Q3W ×6 + oxaliplatin 135 mg/m^2^, D1,	
					Q3W ×6; liver surgery	
Adebrelimab +	NCT06208462	Phase 2, single-center,	Resectable ICC	33	Adebrelimab, 1,200 mg, day 1, Q3W	1-year objective
lenvatinib +		single-arm, open-label			x2-4 + lenvatinib, 8 mg, qd + gemcitabine,	response rate
HAIC GEMOX					1,000 mg/m^2^, Q3W ×2-4 + cisplatin,	
					25 mg/m^2^, Q3W ×2-4 cycle (HAIC)	
Toripalimab +	NCT04669496	Phase 2, 3, multicenter,	Resectable ICC	178	Arm A: Toripalimab, 240 mg, day 1,	2-year event-free
lenvatinib +		randomized, open-label			Q3W ×3 + lenvatinib, 8 mg, qd, 9W +	survival
GEMOX					gemcitabine, 1,000 mg/m^2^, D1 and D8,	
					Q3W ×3 + oxaliplatin 85 mg/m^2^, D1,	
					Q3W ×3; liver surgery. Arm B: Surgery	
Toripalimab +	NCT04506281	Phase 2, multicenter,	Resectable ICC	128	Arm A: Toripalimab, 240 mg, Day 1,	18-month event-free
lenvatinib +		randomized, open-label			Q3W ×3 + lenvatinib, 8 mg, qd, 9W +	survival
GEMOX					gemcitabine, 1,000 mg/m^2^, D1 and D8,	
					Q3W ×3 + oxaliplatin 85 mg/m^2^, D1,	
					Q3W ×3; liver surgery. Arm B: Surgery	
Durvalumab +	NCT06050252	Phase 2, single-center,	Resectable ICC	27	Durvalumab, day 1, Q3W ×4 +	Proportion of patients
gemcitabine +		single-arm, open-label			gemcitabine + cisplatin, D1 and D8,	who complete four
cisplatin					Q3W ×4	cycles of neoadjuvant
						therapy followed by
						surgical resection
Durvalumab +	NCT06017297	Phase 2, single-center,	Borderline	28	Durvalumab, 1,500 mg, day 1, Q3W ×4-8 +	Rate of conversion
tremelimumab +		single-arm, open-label	resectable/		tremelimumab, 300 mg, day 1 (single	from unresectable
gemcitabine +			resectable ICC		dose) + gemcitabine, 1,000 mg/m^2^, D1 and	to resectable and
cisplatin					D8, Q3W ×4-8 + cisplatin 25 mg/m^2^, D1	incidence of treatment-
					and D8, Q3W ×4-8; liver surgery	associated adverse
						events

W, week; qd, once a day; GEMOX, gemcitabine + oxaliplatin; HAIC, hepatic arterial infusion chemotherapy; NCT, National Clinical Trial; ICC, intrahepatic cholangiocarcinoma.

## Data Availability

Not applicable.
